# Using Nature to Nurture: Breast Milk Analysis and Fortification to Improve Growth and Neurodevelopmental Outcomes in Preterm Infants

**DOI:** 10.3390/nu13124307

**Published:** 2021-11-29

**Authors:** Katherine Marie Ottolini, Elizabeth Vinson Schulz, Catherine Limperopoulos, Nickie Andescavage

**Affiliations:** 1 Department of Pediatrics, Division of Neonatal-Perinatal Medicine, Uniformed Services University of the Health Sciences, Bethesda, MD 20814, USA; katherine.ottolini@usuhs.edu (K.M.O.); elizabeth.schulz@usuhs.edu (E.V.S.); 2 Department of Pediatrics, George Washington University School of Medicine, Washington, DC 20037, USA; climpero@childrensnational.org; 3 Department of Radiology, George Washington University School of Medicine, Washington, DC 20037, USA; 4 Developing Brain Research Laboratory, Children’s National Hospital, Washington, DC 20010, USA; 5 Department of Neonatology, Children’s National Hospital, Washington, DC 20010, USA

**Keywords:** preterm, breast milk, fortification, neurodevelopment

## Abstract

Premature infants are born prior to a critical window of rapid placental nutrient transfer and fetal growth—particularly brain development—that occurs during the third trimester of pregnancy. Subsequently, a large proportion of preterm neonates experience extrauterine growth failure and associated neurodevelopmental impairments. Human milk (maternal or donor breast milk) is the recommended source of enteral nutrition for preterm infants, but requires additional fortification of macronutrient, micronutrient, and energy content to meet the nutritional demands of the preterm infant in attempts at replicating in utero nutrient accretion and growth rates. Traditional standardized fortification practices that add a fixed amount of multicomponent fortifier based on assumed breast milk composition do not take into account the considerable variations in breast milk content or individual neonatal metabolism. Emerging methods of individualized fortification—including targeted and adjusted fortification—show promise in improving postnatal growth and neurodevelopmental outcomes in preterm infants.

## 1. Introduction

The third trimester of pregnancy represents a period of rapid fetal growth resulting from increased placental nutrient and energy transfer. The rate of fetal protein accretion during the second half of pregnancy is estimated to be approximately 2 g/kg/day [1,2]. Fat accretion occurs almost entirely after 25 weeks’ gestation, increasing exponentially thereafter and peaking at 7 g/day by term [3]. Adequate nutrient transfer during this timeframe is particularly essential for the developing human brain, with cerebral and cerebellar volumes increasing by 230% and 384%, respectively, between 25- and 37-weeks’ gestation in healthy fetuses [4].

In comparison to the developing fetus, preterm infants born during this critical developmental window are exposed to unique environmental stressors and systemic illness within the extrauterine environment that pose additional nutritional demands to achieve growth rates that parallel in utero nutrient accretion [2]. Despite advances in neonatal nutrition, half of all very low birth weight (VLBW, <1500 g) infants continue to experience extrauterine growth restriction, which has been closely tied to poor neurodevelopmental outcomes [5,6,7]. Postnatal growth has major implications for preterm brain development, as greater increases in weight, linear growth, and head circumference have all been associated with improved long-term neurodevelopment outcomes [8,9].

Substrates for enteral nutrition in these high-risk infants include bovine or human-milk-derived products. Human milk administration has several well-established benefits in this population, conferring protection against common morbidities associated with impaired growth and neurodevelopment—including sepsis, necrotizing enterocolitis, and bronchopulmonary dysplasia [10,11]. Breast milk intake has also been associated with superior brain growth and microstructural development, as well as short and long-term neurodevelopmental outcomes compared to preterm formula, likely as a result of its unique bioactive and nutritional components [11,12]. Exclusive human milk feeding is therefore recommended as the standard of care for preterm infants, with the provision of donor breast milk when the mother’s own milk is not available [10,13]. However, unfortified human milk does not adequately meet the nutritional demands of the growing preterm infant, warranting fortification with additional macro- and micronutrients [10,14]. In this review, we aim to discuss current and evolving methods of breast milk fortification with the aim of optimizing postnatal growth rates and neurodevelopment, including the use of individualized fortification and human milk analysis.

## 2. Human Milk Analysis

### 2.1. Crematocrit

Human milk analysis began in the late 1970s based on a microcentrifugation technique originally used for estimating the fat content in goats’ milk [15]. This method for estimation of human milk’s energy and fat content advertised a “rapid and cheap” analysis of the percentage of cream within the milk, thus named the “*creamatocrit*” [15]. In this process, a homogenized sample of human milk is drawn into a standard capillary tube. The sample is centrifuged and then immediately removed and placed upright, and the layer of fat at the top of the tube is then measured with calipers [15,16]. This value represents a percentage of the total volume of milk in the tube, and an estimation of fat and energy content (kcal/30 mL) may subsequently be derived using the following calculations [14,15,17].

(1) $$\mathrm{fat}\;(g/L)=\frac{\left(creamatocrit\;\left[\%\right]-0.59\right)}{0.146}$$

kcal/L = (290 + 66.8) × *creamatocrit* (%) (2)

Unfortunately, despite the simplicity of obtaining a *creamatocrit*, this tool only provides data for the fat content of the milk. Lucas et al. additionally reported the potential for overestimation of the *creamatocrit*, dependent upon what location along the meniscus (superior versus inferior border) the calipers measured [15]. This concern was confirmed by O’Neill et al., who demonstrated an overestimation of the fat and energy content of human milk using the *creamatocrit* microanalysis method in comparison to a human milk analyzer (mid-infrared spectroscopy method) [16]. With newer technologies, the *creamatocrit* has become a historical means of fat content analysis in human milk but may have utility for late preterm and term infants in low-resource settings when alternate technologies are unavailable. Nevertheless, this practice is cautioned for use in VLBW and extremely low birth weight (ELBW, <1000 g) infants due to their increased nutritional demands and the vast changes in breast milk content that take place over the initial weeks of lactation for their mothers.

### 2.2. Biochemical Methods

Originating from the bovine dairy industry in Europe, several standardized laboratory biochemical approaches to assess macronutrient content in human milk exist, including the Gerber method for fat concentration, the biuret method for protein content, and Marier and Boulet’s phenol-sulphuric acid colorimetric method for lactose content [18,19,20,21,22,23]. In the Gerber method, homogenized milk is combined with sulfuric acid and amyl alcohol with centrifugation to produce separation of the fat [24]. This reaction utilizes a butyrometer, a specialized scaled container that measures the percentage of fat following separation, with each percentage representing a specific volume. For total protein analysis, the biuret assay aims to induce formation of a complex between peptide molecules and copper salts under alkaline conditions [23]. If the biuret reaction occurs, the complex will become violet in appearance and a spectrophotometer is then utilized to measure the absorbance at ~540 nm in order to calculate the concentration of protein within the sample [23]. Lastly, colorimetry may be utilized for determining the lactose content in human milk in which a homogenized milk sample is mixed with phenol-sulphuric acid or a combination of zinc sulfate and barium hydroxide and then centrifuged. The resultant clear supernatant is further processed, and absorbance read at ~520 nm to calculate lactose concentration [25]. It should be noted that alternative biochemical methods are available for the measurement of each of these macronutrients.

### 2.3. Spectroscopy

As with the previous methods of human milk analysis, spectroscopy use originated in the bovine milk industry. Although human milk analyzers, which use either near-infrared or mid-infrared spectroscopy (NIRS/MIRS, respectively), are reported in the literature for targeted nutrition for premature infants since the 1980s, approval by the United States Food and Drug Administration (FDA) for the use of a breast milk analyzer did not occur until 2018 [26,27]. Current human milk analyzers primarily use spectrophotometry to assess the fat, carbohydrate (lactose), protein, and energy content [28]. The principle behind the use of infrared analysis in human milk is the identification of chemical groups (fat, lactose, and protein) through their absorbance of infrared energy [29]. The output from the various analyzers represents each of these components (i.e., macronutrients) based on their wavelength. Near-infrared spectroscopy differs from mid-infrared spectroscopy in the portion of the wavelength spectrum the spectrophotometry is taking place; NIRS utilizes a wavelength spectrum of 1200 to 2400 nm, while MIRS utilizes 1300 to 3000 nm [29].

There is considerable variation in the accuracy of spectroscopy as it compares to traditional biochemical techniques in the dairy industry [16,21]. Much of this variability arises from the multiple types of milk analyzers (e.g., different brands, near-infrared versus mid-infrared) and the variety of biochemical techniques that are utilized globally for the estimation of macronutrients. As such, the comparisons between different types and brands of analyzers differ depending on the various biochemical techniques used. However, when standardization and rigorous calibration are maintained, human milk analyzers provide accurate, reliable, and rapid measurements of macronutrient and energy content [30,31,32].

### 2.4. Breast Milk Content

**Figure 1:**
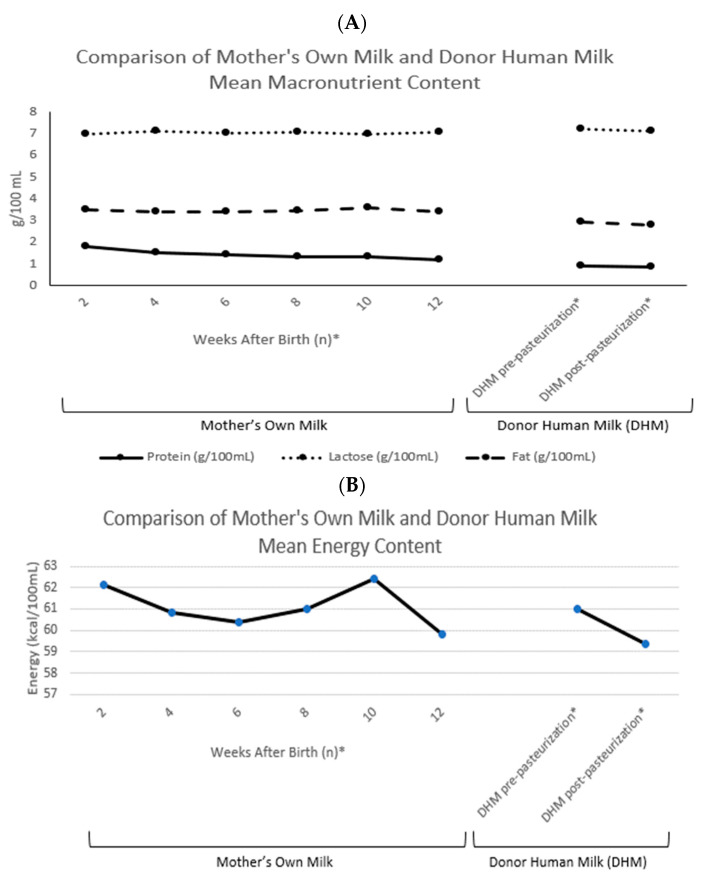
(**A**) Comparison of mother’s own milk and donor human milk mean macronutrient content (protein, lactose and fat; g/100 mL). (**B**) Comparison of mother’s own milk and donor human milk mean energy content. Means extracted from Piemontese et al. and Zachariassen et al. The values presented represent native (unfortified) human milk [37,41]. * Values representing the weeks after delivery are indicative of the mother’s own milk averages, whereas the donor human milk (DHM) averages represent over 90% of donor milk mothers who delivered after 37 weeks’ gestation. Donation began 2.9 ± 2.3 months after delivery [37].

Numerous studies have revealed nutrient variability in human milk from lactating mothers of preterm infants [33,34,35]. Although the estimated energy average of breast milk for the purposes of standardizing fortification is 19–20 kcal/30 mL, human milk is a dynamic fluid that yields varying macronutrient and energy densities depending on time of day, degree of premature delivery, and stage of lactation. Because of these variations, standardization of the fortification process may not meet the individual nutritional needs for optimal growth and neurodevelopment in VLBW and ELBW infants. Maternal colostrum, which is the early, small volume supply of human milk, constitutes the maximum density of protein (reported as g/100 mL) during lactation [36]. As lactation continues, in addition to diurnal variations, the content of protein continues to drop, such that premature human milk resembles that of term milk within weeks of delivery (Figure 1) [33].

When an adequate supply of mother’s own milk is not available, supplementation with pasteurized donor breast milk from an established human milk bank is recommended [10]. In an effort to protect infants of mothers who donate their expressed milk, most milk banks and donor human milk companies will not accept human milk donations until several weeks postpartum and, in many cases, require demonstration of appropriate growth in the mother’s own infant. Therefore, donated human milk products and their macronutrients more often resemble term milk and/or later stages of postpartum lactation—including decreased protein, fat, and energy content as compared to preterm milk [37]. One strategy utilized by international milk banking associations is to pool milk from multiple donors for the collective benefits of variable macronutrient content and a uniform batch of donor milk [38]. Nevertheless, the energy content of even pooled donor milk remains, before and after pasteurization, less than mother’s own milk (Figure 1) [39,40]. Fortification of either mother’s own milk or donor human milk with a bovine milk-derived or human-milk-derived fortifier aims to increase the macro- and micronutrient content to promote optimal growth of the high-risk preterm neonate [13].

## 3. Breast Milk Fortification

### 3.1. Standardized Fortification

Standardized (also known as “fixed-dose” and “blind”) fortification is based on assumed breast milk macronutrient (protein, fat, carbohydrate), micronutrient, and energy content from reference values, wherein mother’s own milk and donor human milk are both estimated at ~20 kcal/30 mL [13]. Typically, a multicomponent fortifier is added to the human milk substrate to achieve a desired energy content between 22–26 kcal/30 mL. As mentioned previously, variations in diurnal and week-to-week maternal milk content throughout lactation bring caution to the assumptions of a standard milk composition. The variable composition of donor human milk, due to inter-donor variations in lactation phase and the impact of pasteurization on nutritional content, raises added concerns (Figure 1). Additionally, the differences between bovine and human-milk-derived fortifiers may affect the ability to tailor the human milk substrate to optimal macronutrient content [13]. A variety of concentrated bovine milk-derived fortifiers exist on the market to not only enhance caloric density, but also improve delivery of higher protein content. Similarly, commercially available human-milk-derived fortifiers incorporate a selection of macronutrient additives with the aim to provide an exclusive human milk or human-milk-derived diet comparable to the bovine milk-derived products (Figure 1) [41].

### 3.2. Individualized Fortification

With the variable content human milk over time, as well as the unique nutritional requirements of individual infants, it is not surprising that standardized fortification may not provide optimal macronutrient content in half of very low birth weight infants [5,42,43]. Nutritional and technological advances, including the development of modular macronutrient fortifiers and the increased availability of breast milk analysis within the neonatal intensive care unit (NICU), have led to a growing wave of ‘lacto-engineering’ and customized nutrition for the high-risk neonate. Strategies for individualized breast milk fortification include adjusted and targeted fortification, as well as combinations of both methods. Results from a recent national survey of U.S. NICUs demonstrated that only 12% of respondents currently utilize human milk analysis, whereas 41% employ adjusted fortification methods and 98% use modular macronutrient products [44].

In targeted fortification, a sample of human milk (via NIRS/MIRS) is analyzed to determine its specific macronutrient and energy content, and then additional macronutrient supplementation is provided to achieve goal values. Targeted values for the macronutrient and energy content of enteral feeds are typically based on consensus recommendations for preterm infants, including 3.5–4.5 g/kg/day protein, 4.8–6.6 g/kg/day lipids, 11.6–13.2 g/kg/day carbohydrates, 110–135 kcal/kg/day, and protein/energy ratios of 3.2–4.1 g/100 kcal [2]. Fortification may be achieved through a combination of multicomponent fortifiers, as well as specific modular macronutrient products such as medium-chain triglycerides, safflower oil, whey protein, casein-based liquid protein, maltrodextrin, and glucose polymers [43,45,46,47,48,49]. A human-milk-derived cream fortifier is also commercially available to provide additional fat, protein, and carbohydrate content [45]. The frequency of human milk analysis in studies of targeted fortification has ranged from twice daily to weekly [43,45,46,48,49,50]. In a study evaluating the effects of differing sampling intervals ranging from daily to weekly, Rochow et al. found that twice weekly milk analysis achieved macronutrient intake within 5% of targeted goals [50]. Although targeted fortification is an appealing method of providing customized nutrition to preterm neonates, the process can be time- and labor-intensive as well as cost-prohibitive. It requires the purchase of a human milk analyzer, of which there is currently only one U.S. FDA-approved device. In addition to equipment expenses, specialized training is also required to properly analyze milk and provide tailored fortification. Coordination with lactating mothers is also necessary to obtain accurate milk samples that are reflective of average macronutrient content. This process may involve large, multidisciplinary care teams including pharmacy or milk laboratory technicians, registered dieticians, lactation consultants, and physicians [44].

Adjustable fortification poses a less labor-intensive approach to individualized fortification, in which neonatal growth velocity along with laboratory markers of protein metabolism are utilized to estimate protein requirements [13]. Blood urea nitrogen (BUN) level is the most commonly utilized laboratory value in this tailored approach, although additional markers that have been cited in the literature include the BUN-to-creatine ratio and corrected serum urea nitrogen (adjusted for the serum creatinine level) [51,52,53,54,55,56]. The majority of published protocols aim for BUN levels ranging from 9–16 mg/dL, although targets as low as >3–5 mg/dL have been utilized [10,51,52,53,54,55,56]. Laboratory values are monitored once or twice weekly and protein fortification is adjusted accordingly utilizing either multicomponent fortifiers or modular protein additives. Recently, the urinary urea-to-creatinine ratio has been explored as a potential non-invasive marker of protein metabolism, demonstrating a high correlation between the urinary urea-to-creatinine ratio, serum BUN levels, and actual protein intake in preterm neonates [57]. In contrast to targeted fortification, adjustable fortification only allows for estimation of an infant’s protein requirements, but it is more easily implemented and titrates protein administration based on an individual neonate’s metabolic response [13].

Some individualized fortification protocols have utilized a combination of targeted and adjustable fortification. In one such approach, the mother’s milk is first analyzed and fortified to achieve preset targeted goals for macronutrient and energy content. Protein supplementation is then further tailored based on laboratory monitoring [58].

## 4. Growth Outcomes

### 4.1. Standardized Fortification

Before the fortification of breast milk in preterm neonates became the standard of care, several small studies were conducted beginning in the 1980s comparing growth rates between infants receiving standardized fortification and unfortified breast milk. In a 2016 Cochrane review of randomized-controlled trials, Brown et al. found evidence supporting increased in-hospital weight gain, length, and head circumference in infants receiving standardized fortification compared to unfortified breast milk, although included studies were characterized as being small with weak methodology [59]. For infants born small-for-gestational-age (SGA), one study suggested a greater positive effect of fortification on growth rates compared to those born appropriate-for-gestational age (AGA) [60]. A recent large umbrella review of breast milk fortification in VLBW infants found evidence that the multicomponent fortification—with the addition of protein and energy (as fat or carbohydrate)—led to significant increases in weight, length, and head circumference [61].

Human-milk-derived fortifiers are an appealing option to provide an exclusive human milk diet to preterm infants [62]. In a recent systematic review of randomized-controlled trials, Ananathan et al. noted significantly lower weight gain infants receiving human-milk-derived fortifiers in comparison to bovine-derived, without any difference in length or head growth between groups [63]. O’Connor et al. also noted slower weight gain in infants receiving human versus bovine-derived fortification, although this difference was no longer significant when weights were converted into z-scores [64]. In a large study of SGA infants, those receiving human-milk-derived fortification exhibited greater length z-scores by hospital discharge than those receiving bovine milk-derived fortification [62].

In evaluating the timing of fortification, one large cohort study found a significant association between earlier fortification and improved in-hospital length and weight gain in neonates receiving both human-milk-derived and bovine-based fortification [65]. However, these results were not replicated in three randomized controlled trials that showed no difference in growth velocities between infants receiving early versus delayed fortification with either human or cow’s milk-derived products [66,67,68].

There is no consensus on the optimal post-discharge feeding regimen for breast milk-fed preterm infants, and few studies have evaluated the use of multicomponent breast milk fortifiers in the outpatient setting. When fortification was provided in 50% of daily feeds for 12 weeks, infants demonstrated significantly greater weight and length at 12 months of age compared to those receiving unfortified milk, with additional benefits in head growth seen in those born <1250 g [64,69]. Similar growth benefits at one year of age were seen in a study that provided fortifier via breast milk ‘shots’ given prior to direct breastfeeding through 2 months corrected age [70]. In contrast, no benefits from fortification were noted when a considerably lower volume of fortifier was administered once a day through 4 months corrected age [71].

### 4.2. Individualized Fortification

Targeted fortification utilizing human milk analysis has been associated with improved growth rates compared to standardized methods [43,45,46]. Studies using a combination of multicomponent fortifier and modular macronutrient supplements have demonstrated superior weight, length, and head circumference in infants receiving targeted fortification [43,46]. One study that achieved targeted fortification utilizing the addition of a human milk-based cream also found superior growth velocities for weight and length compared to standardized fortification [45]. In two studies that did not find any growth benefits from targeted fortification, the macronutrient content of analyzed milk was actually greater than assumed reference values, such that infants in the targeted fortification group received less supplementation compared to controls receiving standardized fortification [48,49].

Recent studies of adjustable fortification strategies utilizing goal BUN values of >3 mg/dL, >5 mg/dL, 9–14 mg/dL, and 10–16 mg/dL have all demonstrated superior growth rates compared to standardized fortification [51,52,53,54,55,56]. Studies of in-hospital growth have revealed improved weight, length, and head circumference in infants receiving adjustable fortification [51,52,56]. Picauld et al. similarly demonstrated improved weight, length, and head circumference z-scores following the implementation of adjustable fortification [53]. Biasini et al. did not observe an overall effect of adjustable fortification on in-hospital growth rates, but infants who received adjustable fortification experienced greater post-discharge length at 9 months corrected age compared to standardized fortification [54]. ELBW infants managed with adjustable versus standardized fortification have demonstrated superior in-hospital growth velocities for weight, length, and head circumference, as well as greater post-discharge head circumference through 24 months corrected age [54,55].

A recent systematic review evaluating growth outcomes following individualized fortification revealed stronger evidence for the growth benefits of adjustable versus targeted fortification based on available studies [72]. One study comparing targeted versus adjustable fortification strategies noted a greater daily increase in weight and head circumference in the targeted group, although a low BUN threshold (>5 mg/dL) was utilized in the adjustable fortification group [73]. A study by Simsek et al. found that both targeted and adjustable fortification methods achieved greater weight and head circumference compared to standardized fortification [74].

## 5. Neurodevelopment

### 5.1. Standardized Fortification

Few studies have specifically evaluated the effects of breast milk fortification on neurodevelopmental outcomes. Lucas et al. did not find any significant differences in Bayley Scale of Infant Development (BSID) scores at 9 and 18 months between infants receiving bovine-derived standardized fortification versus unfortified breast milk. However, these results should be interpreted with caution, as this study was performed more than two decades ago and breast milk comprised less than 50% of milk intake in both groups [75]. Kashaki et al. followed preterm neonates through 3 years of age to evaluate the long-term neurodevelopmental impact of high protein administration versus lower protein administration utilizing a bovine-based multicomponent fortifier. The high-protein group demonstrated greater communication and gross motor scores based on the Ages and Stages Questionnaire, as well as improved development in auditory, verbal language and perception, and cognitive domains using the Newsha Developmental Scale [76].

A comparison of BSID-III scores at 18 months corrected age between human and bovine-milk derived fortification found no significant differences between groups, although investigators acknowledged that the study may not have been powered to detect all clinically-significant differences [77]. Similarly, a study of ELBW infants who received human versus bovine-derived fortification failed to demonstrate any significant differences in BSID III cognitive scores through 18 months corrected age [78]. A potential neuroprotective effect of human versus bovine-derived fortification was noted in a study of ELBW infants, which found that fortification with a human-milk-derived fortifier was associated with a decreased incidence of severe intraventricular hemorrhage or periventricular leukomolacia [79].

Studies of post-discharge fortification have demonstrated modest, if any, neurodevelopmental benefits [69,80,81]. Twice daily supplementation of breast milk with a bovine-based fortifier through 4–6 months corrected age was not associated with any improvements in 12-month BSID-III scores [81]. Fortification of 50% of breast milk feeds with a bovine-based fortifier for 12 weeks was associated with improved visual acuity at 4 and 6 months corrected age, although no significant impact on BSID-III scores was seen at 18 months corrected age [69,80].

### 5.2. Individualized Fortification

Adjustable fortification has been associated with improved neurodevelopmental outcomes through 2 years of age [52,54,55]. Ergenekon et al. found significantly higher BSID-III scores at 18 months corrected age in preterm neonates who had received adjustable versus standardized fortification for both the mental and psychomotor developmental index [52]. Using the Griffiths Mean Developmental Score (GMDS), Biasini et al. demonstrated significantly higher hearing and language scores at 12 and 18 months corrected age in infants receiving adjustable fortification. The greatest developmental benefits of adjustable fortification were seen in the subset of SGA infants, who exhibited superior GDMS scores in nearly all domains at 18 and 24 months corrected age [54]. Those ELBW infants who received adjustable fortification demonstrated higher GDMS performance scores at 3 months and hearing and language scores at 12 months corrected age. In a study comparing ELBW infants managed with the same adjustable fortification protocol to controls receiving standardized fortification, Mariani et al. noted a drop in GMDS scores between 12 and 24 months corrected age in the standardized but not the adjustable fortification group [55].

### 5.3. Future Directions

It is difficult to elucidate the precise impact of early breast milk feeding on long-term neurodevelopment, especially when the decision to breastfeed is highly correlated with a multitude of genetic and environmental factors that may also influence brain development including maternal race/ethnicity, socio-economic status, intelligence quotient, and educational level [82]. Novel quantitative magnetic resonance imaging (MRI) techniques—including volumetric segmentation and diffusion tensor imaging—are emerging as non-invasive methods for evaluating the impact of early nutritional interventions on preterm brain development at the microstructural level [12]. Quantitative MRI has already been utilized to demonstrate the effects of early breast milk, macronutrient, and energy intake on preterm brain development by term-equivalent age, and could potentially be utilized as a future tool in determining ideal breast milk fortification practices [12,82,83,84].

## 6. Conclusions

The evolving practices of individualized human milk fortification through targeted and adjustable methods show promise in improving postnatal growth and neurodevelopmental outcomes in preterm neonates. Additional research is needed to elucidate the ideal fortification method to support adequate postnatal growth and long-term neurodevelopment in this vulnerable population.

## Data Availability

Not applicable.
